# Assessing the Influence of COVID‐19 on the Shortwave Radiative Fluxes Over the East Asian Marginal Seas

**DOI:** 10.1029/2020GL091699

**Published:** 2021-02-02

**Authors:** Yi Ming, Pu Lin, Vaishali Naik, Fabien Paulot, Larry W. Horowitz, Paul A. Ginoux, V. Ramaswamy, Norman G. Loeb, Zhaoyi Shen, Clare E. Singer, Ryan X. Ward, Zhibo Zhang, Nicolas Bellouin

**Affiliations:** ^1^ NOAA/Geophysical Fluid Dynamics Laboratory Princeton NJ USA; ^2^ NASA/Langley Research Center Hampton VA USA; ^3^ Department of Environmental Science and Engineering California Institute of Technology Pasadena CA USA; ^4^ Department of Physics University of Maryland Baltimore County Baltimore MA USA; ^5^ Joint Center for Earth System Technology University of Maryland Baltimore County Baltimore MA USA; ^6^ Department of Meteorology University of Reading, Reading Reading Whitenights UK

## Abstract

The Coronavirus Disease 2019 (COVID‐19) pandemic led to a widespread reduction in aerosol emissions. Using satellite observations and climate model simulations, we study the underlying mechanisms of the large decreases in solar clear‐sky reflection (3.8 W m^−2^ or 7%) and aerosol optical depth (0.16 W m^−2^ or 32%) observed over the East Asian Marginal Seas in March 2020. By separating the impacts from meteorology and emissions in the model simulations, we find that about one‐third of the clear‐sky anomalies can be attributed to pandemic‐related emission reductions, and the rest to weather variability and long‐term emission trends. The model is skillful at reproducing the observed interannual variations in solar all‐sky reflection, but no COVID‐19 signal is discerned. The current observational and modeling capabilities will be critical for monitoring, understanding, and predicting the radiative forcing and climate impacts of the ongoing crisis.

## Key Points

- Solar clear‐sky reflection was observed to drop substantially over the East Asian Marginal Seas in March 2020
- Climate model simulations nudged with reanalysis data are used to separate the impacts of meteorology and emissions
- It is found that about one‐third of the clear‐sky anomalies can be attributed to pandemic‐related emission reductions

## 1 Introduction

The lockdown measures instituted to control the spread of Coronavirus Disease 2019 (COVID‐19) caused unprecedented disruptions to many economic sectors, among which manufacturing and transportation were particularly hard hit. The consequent decrease in emissions of anthropogenic aerosols and their precursors generally led to improvements in air quality and visibility (Mahato et al., 2020; McNeill, 2020; Sharma et al., 2020), with notable exceptions (Le et al., 2020). These emission reductions may have had an influence on Earth's radiation budget, and by extension weather and climate, as short‐lived aerosol particles have long been postulated to provide a net cooling by scattering/absorbing insolation (direct effects) under clear‐sky conditions (Bellouin et al., 2003; Haywood, 1999; Mitchell, 1971) and brightening clouds (indirect effects) under cloudy conditions (Albrecht, 1989; Twomey, 1974).

**Figure 1:**
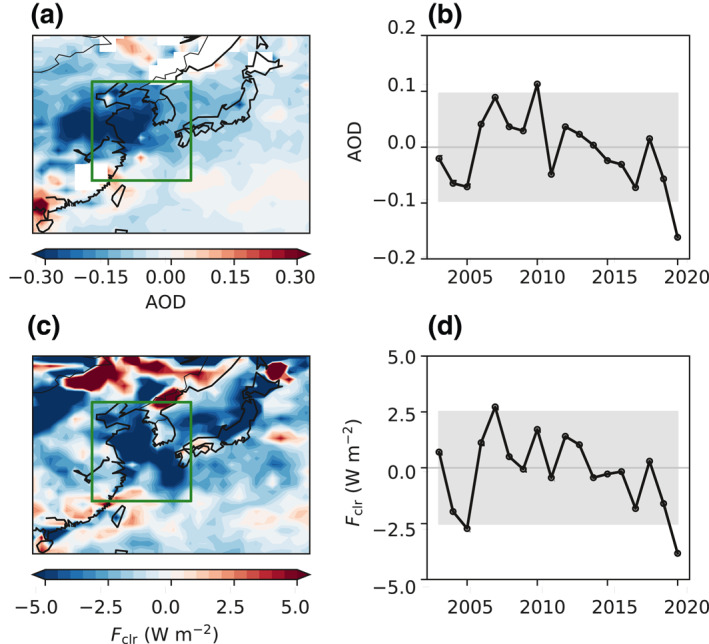
(a) Spatial distribution of the anomaly in MODIS aerosol optical depth (AOD) in March 2020. The oceanic region enclosed by the green rectangle (117°–132°E and 26°–41°N) is defined as the East Asian Marginal Seas (EAMS). (b) Time series of the anomaly in MODIS AOD over EAMS in March from 2003 to 2020. The gray area denotes the 90% confidence interval over the climatological period. (c) Same as (a), but for CERES shortwave clear‐sky top‐of‐atmosphere (TOA) radiative flux (*F*_clr_, upward defined as positive). (d) Same as (b), but for CERES *F*_clr_. The climatology is defined as 2003–2019. MODIS, Moderate Resolution Imaging Spectroradiometer; CERES, Clouds and the Earth's Radiant Energy System.

Satellite observations offer some indications. In March 2020, one month after China implemented a strict lockdown, the Moderate Resolution Imaging Spectroradiometer (MODIS) aerosol optical depth (AOD) (Remer et al., 2005) exhibited large negative anomalies relative to the climatological (2003–2019) mean, not only over much of East Asia, but also extending downwind over the Pacific (Figure 1a). The average decrease over the East Asian Marginal Seas (EAMS) (defined as the oceanic region in 117°–132°E and 26°–41°N) was 0.16 W m^−2^, or 32% of the climatological mean (Figure 1b). We choose EAMS as the main analysis region for its proximity to the upwind source regions, more reliable satellite retrievals over ocean than over land (Hsu et al., 2013), and absence of surface snow/ice cover. The concurrently measured Clouds and the Earth's Radiant Energy System (CERES) Energy Balanced and Filled (EBAF) shortwave clear‐sky top‐of‐atmosphere (TOA) radiative flux (*F*_clr_, upward defined as positive; Loeb et al., 2018) was also greatly reduced during March 2020 (Figure 1c). The average decrease over EAMS was 3.8 W m^−2^, or 7% (Figure 1d). Both anomalies exceed their respective 90% confidence intervals (Figures 1b and 1d), and the two quantities are strongly correlated on interannual timescales. This suggests that a substantial emissions reduction, presumably caused by COVID‐19, gave rise to lower aerosol loading, resulting in more solar absorption by Earth's surface.

There are, however, inherent difficulties in interpreting the observations. Besides emissions, meteorology plays a prominent role in modulating AOD and *F*_clr_, especially outside of source regions, via multiple pathways (e.g. long‐range transport, hygroscopic growth, and wet removal). For instance, the negative anomalies over EAMS in March 2005, when there was no anomalous emissions reduction, were comparable to those in March 2020 (Figures 1b and 1d). Therefore, a confident attribution of the observed decreases in AOD and *F*_clr_ to the emissions reduction hinges on a reliable approach for isolating the non‐COVID‐19 factors. It is even more challenging to discern possible impacts on shortwave all‐sky TOA radiative flux *F*_all_ due to the complexities involving clouds. This study addresses these issues with a set of climate model simulations forced with known meteorological conditions.

## 2 Methods

### 2.1 Satellite Observations

We use the observed shortwave TOA fluxes and cloud fraction from the CERES project. Observational data for aerosol and cloud properties are retrieved from the MODIS instrument aboard NASA's Aqua satellite. While similar products are available from NASA's Terra satellite, others have reported the degradation of the on‐board MODIS instrument over time, specifically with respect to the cloud properties of interest in this work (Malavelle et al., 2017; Polashenski et al., 2015). All data are Level 3 (L3) monthly products from MODIS Collection 6.1. The L3 monthly product (MYD08_M3) are gridded to 1° by 1° and derived from the daily products (MYD08_D3). The AOD, cloud fraction, cloud effective radius (*R*_e_), and liquid water path (LWP) are retrieved from the MYD08_M3 data set. AOD is derived from the combined Dark Target and Deep Blue AOD at 0.55 *μ*m over the land and ocean. LWP is retrieved from the 3.7 *μ*m band and represents in‐cloud properties. To compare with model outputs, the in‐cloud LWP is converted to a grid‐box mean LWP by multiplying the in‐cloud LWP by the liquid cloud fraction (calculated from the mean cloud fraction and cloud phase properties). The observational data are interpolated to the AM4 grid for analysis.

### 2.2 Model Simulations

We conduct a suite of nudged simulations from January 2000 to April 2020 with the GFDL AM4 (Zhao et al., 2018), which participated in the World Climate Research Program (WCRP) Coupled Model Intercomparison Project Phase 6 (CMIP6) (Eyring et al., 2015) and forms the basis of a climate prediction system (Delworth et al., 2020). The model horizontal winds, temperature, and surface pressure are nudged to the 3‐hourly averaged products from the MERRA‐2 reanalysis (Gelaro et al., 2017) with a nudging time scale of 6 h, as opposed to generating its own meteorology (typical of climate simulations). Still, aerosols, water vapor, and clouds are computed interactively and subject to the same dynamical and physical processes as in a free‐running simulation, posing a stringent test for model physics. The simulations use the monthly sea surface temperatures (SST) and sea ice concentrations prepared for the CMIP6 historical AMIP simulations (Taylor et al., 2000), which are extended to 2020 using the NOAA Optimum Interpolation (OI) SST V2 data (Reynolds et al., 2002). Aerosol concentrations are calculated interactively based on their emissions, chemistry, advection, and dry and wet deposition.

The SO_2_ and black carbon (BC) emissions used in the control simulations are based on the regional Multiresolution Emission Inventory for China (MEIC) (Zhang et al., 2009) in China for 2000–2015 and the CMIP6 historical emissions (Hoesly et al., 2018) in the rest of the world for 2000–2014. The latter is not used for China, because it severely underestimates the decline of SO_2_ after 2007 (Paulot et al., 2018). (Note that MEIC ends in 2015.) The SO_2_ and BC emissions for 2019 are derived by linearly interpolating the CMIP6 SSP (Shared Socioeconomic Pathway) 585 emission scenario between 2015 and 2020 (O'Neill et al., 2016). Emissions for 2016–2018 are derived by interpolating between 2015 and 2019, and those for 2020 are kept as given by SSP585 for the control simulation. Organic matter (OM) emissions (primary OM only) are based solely on the CMIP6 historical and SSP585 inventories. The time evolution of anthropogenic emissions over China is depicted in Figure S1 in the Supporting Information. After peaking in 2007, SO_2_ has been decreasing steadily due to air pollution control measures, while black carbon (BC) and organic matter (OM) diverged after 2015, compensating each other to some extent. Three perturbation simulations are created by reducing the anthropogenic SO_2_, BC, and OM emissions over China for February, March, and April 2020 by 20%, 40%, or 60% to mimic the effects of COVID‐19 lockdown. Note that this broad‐brush sensitivity study assumes uniform emissions reduction in various emission factors over China and complements more detailed analyses (Forster et al., 2020; Huang et al., 2020). All other forcings (such as greenhouse gases, solar irradiance, and stratospheric ozone) are based on the CMIP6 historical forcings (Eyring et al., 2015) for 2000–2014 and the CMIP6 SSP585 forcings (O'Neill et al., 2016) for 2015–2020.

## 3 Results

**Figure 2:**
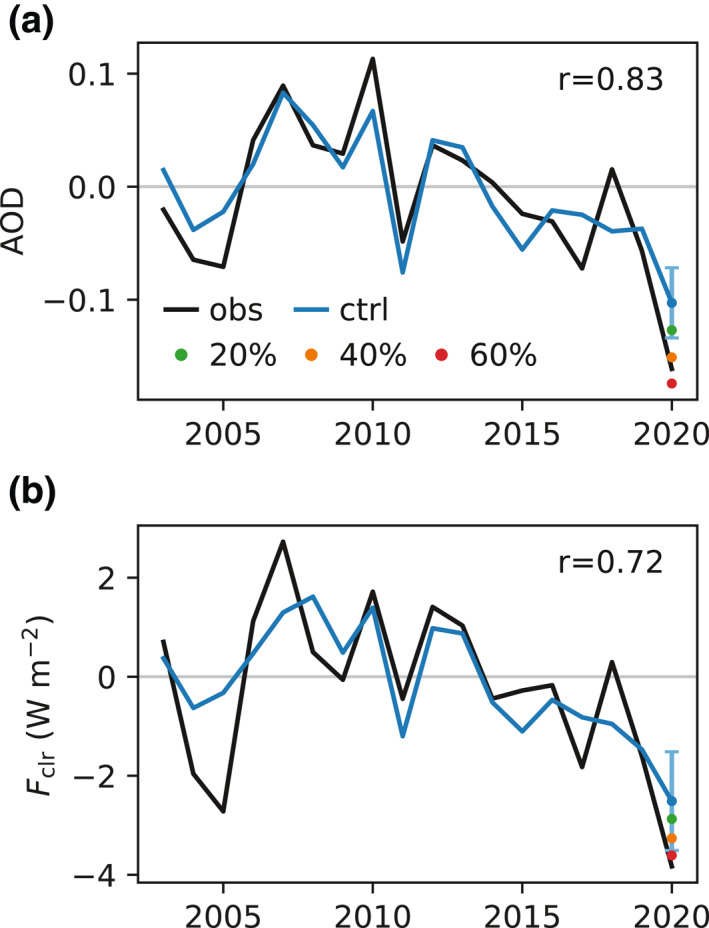
(a) Times series of the anomaly in AOD over EAMS in March from 2003 to 2020. The black line is from MODIS, and the blue line is from the control simulation. The vertical bar denotes the detection limit (one standard deviation of the differences between the observations and the control simulation from 2003–2019). The green, orange, and red dots denote the perturbation simulations of 20%, 40%, and 60% emissions reductions, respectively. *r* is the correlation coefficient. (b) Same as (a), but for CERES *F*_clr_. AOD, aerosol optical depth; MODIS, Moderate Resolution Imaging Spectroradiometer; CERES, Clouds and the Earth's Radiant Energy System. EAMS, East Asian Marginal Seas.

**Figure 3:**
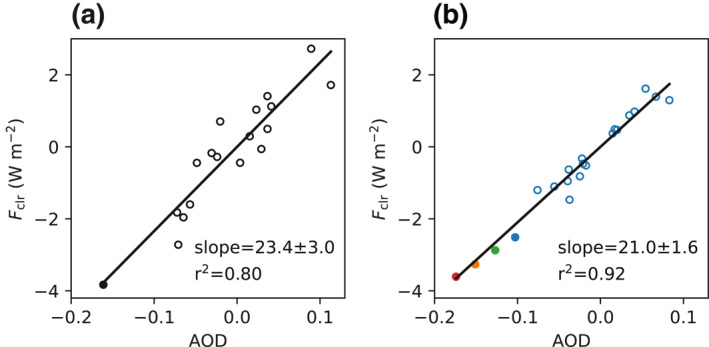
Scatter plots of the anomalies in AOD and *F*_clr_ in March over EAMS. Open dots represent the climatological period (2003–2019) and solid dots represent the year 2020. (a) Observations from MODIS and CERES. (b) Blue dots are from the control model simulation. Green, orange, and red dots correspond to the 20%, 40%, and 60% perturbation simulations, respectively. The regression line is calculated for the climatological period (2003–2019). AOD, aerosol optical depth; MODIS, Moderate Resolution Imaging Spectroradiometer; CERES, Clouds and the Earth's Radiant Energy System.

The nudged control simulation shows considerable skill in reproducing the observed interannual variations of AOD and *F*_clr_ for March over EAMS; the correlation coefficients (*r*) between model and observations are 0.83 and 0.72, respectively (Figure 2). The model performance is comparable in February and April (Figure S2). This suggests that the nudged AM4 simulations provide an effective way to quantify the non‐COVID‐19 influence. The most notable deficiency is that the simulation does not capture the full extent of the negative anomalies in March 2005. However, the model‐simulated AOD and *F*_clr_ anomalies are strongly correlated (Figure 3b), with a slope that is very close to the observationally based counterpart (Figure 3a). This supports the fidelity of the model's representation of the aerosol direct effects. Both observed anomalies emerge from the lower bounds of the detection limits, meaning that they are likely to contain forced components; likelihood is 92% for AOD and 80% for *F*_clr_. The anomalies are estimated at −0.06 for AOD (1.9 standard deviations) and −1.3 W m^−2^ for *F*_clr_ (1.3 standard deviations) by subtracting the control values from the respective observations. When compared with the perturbation simulations, the observations are consistent with a 40%–60% anthropogenic emissions reduction over China (relative to 2020), which roughly translates into a reduction of 31%–47% in SO_2_ emissions relative to 2015, as the baseline SO_2_ emissions in 2015 are 28% higher than in 2020 (Figure S1a). Further, it is important to note that this top‐down estimate is obtained in a way that is fundamentally different from, but complementary to, conventional bottom‐up approaches based on socioeconomic data. One study of the latter kind (Forster et al., 2020) suggests that SO_2_ emissions over China decreased by about 20% in March 2020 (relative to 2015). Given the complicated nature of producing such bottom‐up estimates, it is not expected that they should agree perfectly with our result. It may help reconcile the difference between the two types of estimates to take into account the precise spatiotemporal pattern of the emissions reduction, once known.

**Figure 4:**
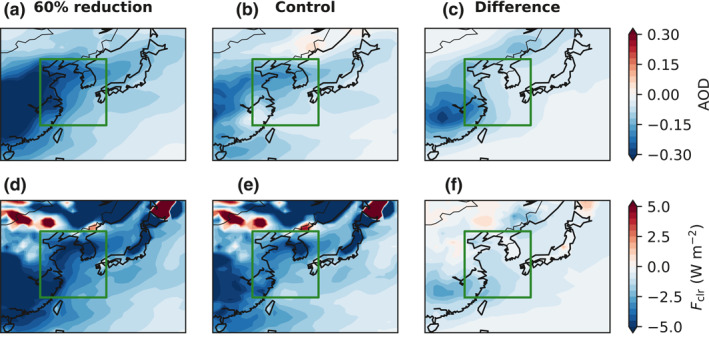
(a) Spatial distribution of the anomaly in AOD in March 2020 from the 60% perturbation simulation. The green rectangle denotes EAMS. (b) Same as (a), but for the control simulation. (c) The difference between (a) and (b). (d)–(f) Same as (a)–(c), but for simulated *F*_clr_. The climatology is defined as 2003–2019 in the control simulation. AOD, aerosol optical depth; EAMS, East Asian Marginal Seas.

We choose the 60% perturbation simulation to illustrate the spatial distributions of the model‐simulated AOD and *F*_clr_ anomalies in Figure 4 (Figure S3 is the same plot for the 40% perturbation simulation). The simulation exhibits a clear land‐sea contrast; the large AOD anomaly over mainland China decreases gradually down the prevailing southwester lies over the ocean (Figure 4a). This pattern is in broad agreement with MODIS (Figure 1a). The overall anomaly can be decomposed into the part due to both the meteorology and long‐term emission trends (non‐COVID‐19) and into the part due to the COVID‐19‐related emissions reduction. The former is the anomaly in the control simulation (Figure 4b), and the latter is the difference between the 60% perturbation and control simulations (Figure 4c). The two contributors to the overall anomaly are of comparable magnitudes, but show different spatial patterns. For instance, the plume cutting across northern China, the Korean Peninsula, and Northern Japan in the non‐COVID‐19 component is not present in the COVID‐19 counterpart. The impact of COVID‐19 on AOD is concentrated over Southern China. These features largely carry over to *F*_clr_ (Figures 4d–4f). The aforementioned decomposition yields insights into the physical mechanisms of regional anomalies. An example is the dipole structure immediately north of the northern boundary of EAMS (41°N), characteristic of the large positive anomalies over parts of Inner Mongolia and Mongolia and the negative anomalies over Northeast China. It can be attributed to meteorology as it exists only in the non‐COVID‐19 component, realized through land surface albedo changes caused by snow melting or accumulation (not shown). A notable discrepancy is that the model projects a large decrease in *F*_clr_ over much of China (Figure 4d), which is not found in the CERES observations (Figure 1c). Although the underlying cause is not entirely clear, it is difficult to reconcile the substantial decrease in MODIS AOD over northern China with the lack of any significant change in CERES *F*_clr_ over the same region given the strong correlation between them (Figure 3). On the other hand, the model substantially overestimates the decrease in AOD and *F*_clr_ over Southern China, but for different reasons. The former is due to the COVID‐19‐related emissions reduction, while the latter belongs to the non‐COVID‐19 component. This seems to suggest that the emissions reduction over Central China may have been overestimated (Figures S9–S11).

**Figure 5:**
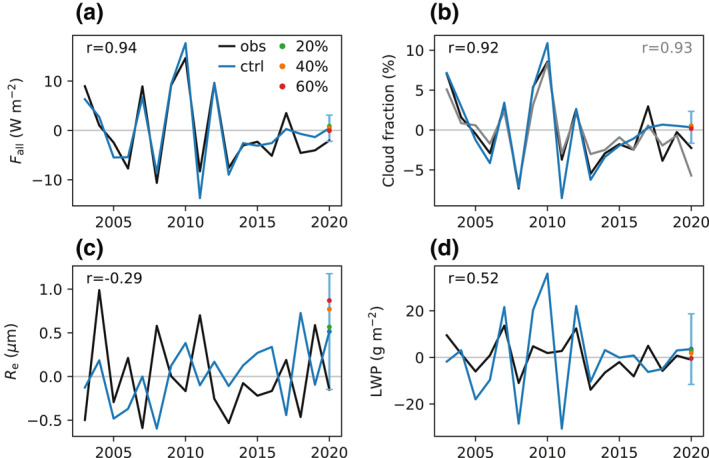
(a) Time series of the anomaly in shortwave all‐sky TOA radiative flux (*F*_all_) over EAMS in March from 2003 to 2020. The black line is from CERES, and the blue line is from the AM4 control simulation. The vertical bar denotes the detection limit. The green, orange, and red dots denote the perturbation simulations of 20%, 40% and 60% emissions reductions, respectively. *r* is the correlation coefficient. (b) Same as (a), but for cloud fraction. The black line is from CERES, and the gray line is from MODIS. The detection limit is based on CERES. (c) Same as (a), but for cloud effective radius (*R*_e_). (d) Same as (a), but for liquid water path (LWP). In (c) and (d) the observation is from MODIS. MODIS, Moderate Resolution Imaging Spectroradiometer; CERES, Clouds and the Earth's Radiant Energy System; TOA, top‐of‐the‐atmosphere; EAMS, East Asian Marginal Seas.

Excellent agreement (*r* = 0.94) is seen between CERES and AM4‐simulated shortwave all‐sky flux (*F*_all_) (Figure 5a). This result is somewhat counterintuitive since *F*_all_ is heavily influenced by clouds, which GCMs historically have struggled to simulate owing to the intrinsic difficulties in representing the effects of cloud‐scale turbulence in coarse‐resolution models. We cross‐check this result by comparing the modeled cloud fraction with CERES observations (Figure 5b). The equally impressive model skill (*r* = 0.92) affirms the prominent role of atmospheric motion in dictating cloud fraction and the quality of AM4's cloud scheme. (More work is needed to better understand the contributions from different cloud types.) The negative anomaly in *F*_all_ (−2.1 W m^−2^) for March 2020 is just within the detection limit, while the negative anomaly in cloud fraction is barely outside. Interestingly, MODIS cloud fraction shows a much larger negative anomaly than its CERES counterpart (Figure 5b). Although the sign of the model‐simulated forced signal in *F*_all_ (less reflection) in the 60% perturbation simulation is consistent with our expectation for a COVID‐19‐related emissions reduction, the magnitude (−0.5 W m^−2^) is well within the detection limit. One may interpret this discrepancy as an indication that the model underestimates the strength of the aerosol indirect effects since the clear‐sky results substantiate the scale of the emissions reduction and the realism of the direct effects. There is, however, no clear signal in MODIS‐retrieved cloud effective radius (*R*_e_) (Figure 5c) or LWP (Figure 5d). In summary, our all‐sky analyses indicate that the observed negative anomaly in *F*_all_ for March 2020 was likely caused by weather variability. While nominally consistent with the all‐sky radiative impacts of an emissions reduction, it was realized through lower cloud fraction, instead of higher *R*_e_ or lower LWP, the two main pathways through which the aerosol indirect effects manifest in GCMs (including AM4).

To test the sensitivity to the definition of the analysis region, we more than double the original domain by expanding beyond EAMS further to the open ocean (the oceanic region in 117°–150°E and 26°–41°N). The results are qualitatively the same (Figures S4 and S5). In fact, the model performs better in terms of the interannual variations of AOD and *F*_clr_, reflecting the good agreement between the observed and simulated spatial structures (Figures 1 and 4). A series of additional simulations are conducted to assess the robustness of the key findings. They cover the long‐term emission trends and locations and speciation of the emissions reduction. Although quantitative differences exist, the main conclusions remain valid (see Figures S6–S14 and Text S1).

The above analyses are also performed for February and April (Figures S15–S18). The MODIS AOD in February 2020 is the lowest since 2005 (Figure S15). The control simulation projects a negative anomaly in 2020, but of only half of the observed magnitude. The discrepancy can be accounted for by a 20%–40% emissions reduction. In terms of *F*_clr_, the model is less skillful for February than for March, resulting in a larger detection limit. Unlike AOD, the observed *F*_clr_ falls within the limit. Note that the observed *F*_clr_ is not nearly as variable as the observed AOD in the few years before 2020, breaking the tight linkage between the two quantities for March (Figure 3a). Since the physics governing the AOD‐*F*_clr_ relationship is simple and robust, more needs to be done to reconcile the two retrievals. One possibility is compensation between scattering and absorbing aerosols. Both the observed *F*_all_ and cloud fraction anomalies are smaller than those in the control simulation (qualitatively similar to March), but within their respective detection limits (Figure S16).

Any sign of AOD decrease is gone by April. Although the MODIS AOD is anomalously low in April 2020, the fact that it is very close to the control suggests no significant COVID‐19‐related emissions reduction (Figure S17). This inference is supported by the observed *F*_clr_, which is slightly above the upper bound of the detection limit, opposite to the perturbation simulations. In stark contrast, the observed *F*_all_ shows an outsized negative anomaly of −18.1 W m^−2^, the largest in the entire CERES data record (Figure S18). This coincides with the largest decrease in CERES cloud fraction. The control simulation captures the timing and magnitude of both anomalies, allowing us to attribute them to the specific meteorological conditions in April 2020, as opposed to the anthropogenic aerosol effects. The above findings are consistent with a recent study of CO_2_ emissions during COVID‐19 (Le Quéré et al., 2020), which suggests that the emissions over China decreased substantially in February and March 2020, but almost fully recovered by April. If one assumes that there is no emissions reduction after April, the annual mean change in *F*_clr_ over EAMS in the 40% emissions reduction simulation (−0.19 W m^−2^) is similar to that in the FAST simulation in Yang et al. (2020), where SO_2_ and BC emissions over China are reduced by about 20%–30%. Note that in Yang et al. (2020), aerosol emissions in other parts of the world are reduced after March, a factor that is not considered in this study.

## 4 Discussion and Conclusions

The COVID‐19 pandemic provides an opportunity to evaluate the model representation of the aerosol‐cloud‐radiation interactions, a major source of uncertainty in global weather and climate modeling. The observational evidence for aerosol direct effects is unequivocal, and their model representation is satisfactory. In contrast, it is more difficult to draw definitive conclusions about aerosol‐cloud interactions and indirect effects from the observed shortwave all‐sky flux. This is fundamentally due to the highly variable, fine‐scale nature of clouds, the challenges in retrieving cloud properties on the observational side, and in parameterizing subgrid cloud processes on the modeling side. Nonetheless, the fact that both the model‐simulated perturbations and the observations stay within the detection limits leads us to conclude that there is no evidence suggesting that the model‐simulated aerosol indirect effects are too strong. The observations underline the dominant role of cloud fraction in determining the all‐sky flux. Any attempt at discerning the manifestation of the aerosol indirect effects through cloud microphysical properties (such as *R*_*e*_ and LWP) is contingent on separating out interference from the synoptic‐scale variations in cloud fraction.

Running in the nudged mode to separate the effects of meteorology from emissions, AM4 is skillful at reproducing the observed interannual variations in shortwave TOA radiative fluxes, clear‐ and cloudy‐sky alike. This allows us to distinguish forced signal from weather variability, a prerequisite for interpreting observations. We find that about one‐third of the observed decrease in shortwave clear‐sky reflection over East Asian Marginal Seas (1.3 out of 3.8 W m^−2^ locally) in March 2020 was likely caused by COVID‐19‐related emissions reduction. On the other hand, the concurrent decrease in shortwave all‐sky reflection (2.1 W m^−2^) is within the detection limit, and thus is thought to be caused mainly by weather variability. By leveraging the latest observational and modeling capabilities, the framework described here is ideal for studying the radiative impacts of the ongoing COVID‐19 pandemic, and the resulting perturbations to the energy balance, in other parts of the world (such as Europe and North America).

## Supporting information

Supporting Information S1

## Data Availability

The AQUA/MODIS MYD08 L3 Global 1 Deg. data set was acquired from the Level‐1 and Atmosphere Archive and Distribution System (LAADS) Distributed Active Archive Center (DAAC), located in the Goddard Space Flight Center in Greenbelt, Maryland (https://ladsweb.nascom.nasa.gov/). The CERES data were obtained from the NASA Langley Research Center Atmospheric Science Data Center (https://doi.org/10.5067/TERRA-AQUA/CERES/EBAF-TOA_L3B004.1). Primary AM4 simulation results that may be used to produce the plots are available are available online (https://data.caltech.edu/records/1666).
