# Supplementary material for: Assessing the Influence of COVID‐19 on the Shortwave Radiative Fluxes Over the East Asian Marginal Seas
Source: Geophys Res Lett. 2021 Feb 2;48(3):e2020GL091699. doi: 10.1029/2020GL091699 (PMC7883069; doi:10.1029/2020GL091699)
Supplement: Supplementary file 1 — Supporting Information S1 [file GRL-48-e2020GL091699-s001.docx]

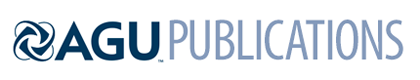


*Geophysical Research Letters*

Supporting Information for

**Assessing the influence of COVID-19 on the radiative balance over the East Asian Marginal Seas**

Yi Ming^1^, Norman G. Loeb^2^, Pu Lin^1^, Zhaoyi Shen^3^, Vaishali Naik^1^, Clare E. Singer^3^, Ryan X. Ward^3^, Fabien Paulot^1^, Zhibo Zhang^4,5^, Nicolas Bellouin^6^, Larry W. Horowitz^1^, Paul A. Ginoux^1^, V. Ramaswamy^1^.

^1^NOAA/Geophysical Fluid Dynamics Laboratory,

^2^NASA/Langley Research Center

^3^Department of Environmental Science and Engineering, California Institute of Technology

^4^Department of Physics, University of Maryland Baltimore County

^5^Joint Center for Earth System Technology, University of Maryland Baltimore County

^6^Department of Meteorology, University of Reading

**Contents of this file**

Text S1 to S3

Figures S1 to S18

Text S1.

As explained in the main text, the anthropogenic emissions over China have been varying non-monotonically in the last two decades, and despite our best effort, there are bound to be inherent uncertainties in the historical emission inventory used in the control simulation. To assess the implications, we re-run the control simulation with the emissions fixed at the 2000 levels. Doing so seriously degrades the model skills for AOD and $F_{clr}$, as the simulations clearly miss the observed long-term trends. These deficiencies tend to inflate the magnitudes of the forced signals for March 2020 (Figs. S6 and S7).

Text S2.

The lockdown first started in Hubei Province before spreading to other parts of China. To study how the locations of the emission reduction affects the results, we divide the country roughly into northern China (NC, north of 35°N), central China (CC, 25­­­°–35°N, where Hubei is located) and southern China (SC, south of 25°N), and reduce the emission by 60% for each region independently. AOD and $F_{clr}$ are more sensitive to the reductions in NC and CC than in SC (Figure S8). The same is true for $F_{all}$ and cloud properties (Figure S12). The spatial distributions are given in Figs. S9-11.

Text S3.

In the perturbation simulations, all three anthropogenic aerosol emissions, namely SO_2_, BC. and OM, are reduced simultaneously. In a set of sensitivity experiments, we reduce one species by 60% at a time. SO_2_ contributes the most to the reductions in AOD and $F_{clr}$. The effects of BC and OM on $F_{clr}$ roughly cancel (Figure S13). $R_{e}$ increases most prominently in the SO_2_ case (Figure S14).

**Figure S1.** Time series of monthly-mean anthropogenic emissions of (a) SO_2_, (b) black carbon (BC), and (c) organic matter (OM) over China.

**Figure S2.** Time series of monthly anomalies in (a) AOD, (b) $F_{\mathrm{clr}}$, and (c) $F_{\mathrm{all}}$. The black and blue lines represent the observations and the control simulation, respectively. The dotted and solid lines represent the monthly anomalies and 11-month running means, respectively. $r$ is the correlation coefficient.

**Figure S3.** Same as Figure 4, but for the 40% perturbation simulation.

**Figure S4.** Same as Figure 2, but for the expanded oceanic region from 117°–150°E and 26°–41°N.

**Figure S5.** Same as Figure 5, but for the expanded oceanic region from 117°–150°E and 26°–41°N.

**Figure S6.** Same as Figure 2, but for the constant emission simulation (the purple line). The blue line is from the control simulation. $r$ is the correlation coefficient for the constant emission simulation.

**Figure S7.** Same as Figure 5, but for the constant emission simulation (the purple line). The blue line is from the control simulation. $r$ is the correlation coefficient for the constant emission simulation.

**Figure S8.** Same as Figure 2, but for the emission location simulations. NC, CC, and SC represent northern China (north of 35°N), central China (25­­­°–35°N) and southern China (south of 25°N), respectively. The regional emissions reduction is 60%.

**Figure S9.** Same as Figure 4, but for the northern China (NC) emission location simulation. The regional emissions reduction is 60%.

**Figure S10.** Same as Figure 4, but for the central China (CC) emission location simulation. The regional emissions reduction is 60%.

**Figure S11.** Same as Figure 4, but for the southern China (SC) emission location simulation. The regional emissions reduction is 60%.

**Figure S12.** Same as Figure 5, but for the emission location simulations. NC, CC, and SC represent the northern China (north of 35°N), central China (25­­­°–35°N) and southern China (south of 25°N), respectively. The regional emissions reduction is 60%.

**Figure S13.** Same as Figure 2, but for the emission speciation simulations. The emission reduction is 60%.

**Figure S14.** Same as Figure 5, but for the emission speciation simulations. The emission reduction is 60%.

**Figure S15.** Same as Figure 2, but for February.

**Figure S16.** Same as Figure 5, but for February.

**Figure S17.** Same as Figure 2, but for April.

**Figure S18.** Same as Figure 5, but for April.
